# The role of mitophagy in female reproductive system diseases: from molecular mechanisms to therapeutic strategies

**DOI:** 10.3389/fendo.2025.1645711

**Published:** 2025-11-18

**Authors:** Huiyi Zhao, Ying Wang, Han Han, Yue Jiang, Xiang Ji, Yuehui Zhang

**Affiliations:** 1 First Clinical Medical College, Heilongjiang University of Chinese Medicine, Harbin, China; 2 Department of Obstetrics and Gynecology, Key Laboratory and Unit of Infertility in Chinese Medicine, First Affiliated Hospital, Heilongjiang University of Chinese Medicine, Harbin, China; 3 Department of Internal Medicine, First Affiliated Hospital, Heilongjiang University of Chinese Medicine, Harbin, China; 4 Department of Gynecology, First Affiliated Hospital, Harbin Medical University, Harbin, China; 5 Department of Physiology/Endocrinology, Institute of Neuroscience and Physiology, The Sahlgrenska Academy, University of Gothenburg, Gothenburg, Sweden

**Keywords:** mitophagy, mitochondrial function, mitochondrial quality control, female reproductive, female reproductive dysfunction

## Abstract

Mitophagy is a catabolic mechanism that selectively degrades damaged mitochondria and precisely modulates mitochondrial content, thereby maintaining intracellular homeostasis under stress conditions. To date, most reviews on mitophagy have predominantly focused on neurodegenerative diseases, cardiovascular disorders, cancer, metabolic syndromes, and inflammation- or immune-related diseases. In recent years, accumulating evidence has highlighted the critical involvement of mitophagy in various physiological and pathological processes associated with female reproduction. This review systematically synthesizes existing evidence to elucidate the regulatory roles of mitophagy during the occurrence and development of follicles, oocyte fertilization, and embryo implantation, as well as its essential contributions to the pathogenesis of endometriosis, polycystic ovary syndrome, primary ovarian insufficiency, and ovarian aging. Furthermore, we outline current therapeutic strategies targeting mitophagy while emphasizing the potential value of traditional Chinese medicine. Our aim is to provide novel insights into the regulatory network and specific targets of mitophagy in female reproduction, facilitate clinical translation, and offer innovative approaches for managing female reproductive health.

## Introduction

In 1963, the Belgian cell biologist Christian de Duve coined the term “autophagy” to describe the cellular process wherein membrane-bound vesicles engulf cytoplasmic components. In the 1990s, Yoshinori Ohsumi and his team successfully identified key autophagy-related genes, and the subsequent cloning of the autophagy-related gene 1 marked a pivotal advancement in autophagy research. Following this breakthrough, the characterization of autophagy genes in mammals was achieved (1–3). Autophagy is a ubiquitous process in eukaryotic cells and can be categorized into macroautophagy, microautophagy, and chaperone-mediated autophagy, all of which play critical roles in maintaining cellular and tissue homeostasis. The form of autophagy primarily discussed in this review is macroautophagy. The discovery of the autophagy receptor sequestosome-1 (SQSTM1/P62) (4) established autophagy as a highly selective recycling mechanism. Depending on the specific molecules and subcellular components targeted for lysosomal degradation and recycling, autophagy can be further subdivided into specialized forms such as mitophagy, ribophagy, reticulophagy, and lipophagy (5). Mitochondria, as double-membrane-bound organelles ubiquitous in eukaryotic cells, are responsible for generating substantial amounts of adenosine triphosphate, which is essential for cellular energy metabolism. Moreover, mitochondria are involved in various critical cellular processes, including fatty acid synthesis, amino acid metabolism, calcium homeostasis, innate immune responses, and apoptosis regulation (6). Mitophagy represents a fundamental mechanism for preserving mitochondrial function and homeostasis by selectively targeting and degrading damaged mitochondria, thereby ensuring the integrity and quality of the mitochondrial population (7–9). In the context of the reproductive system, a sequence of highly coordinated molecular pathways govern sequential stages, encompassing gametogenesis, fertilization, pre-implantation embryo development, embryo implantation, and post-implantation development (10). As the primary energy providers for the ovaries and uterus, mitochondria and their associated autophagic mechanisms are vital to these processes. An increasing body of evidence (11–13) suggests that the mitophagy pathway is intricately involved in key reproductive physiological processes, such as follicular development, fertilization, and implantation, and is closely linked to the pathogenesis of various female reproductive disorders, including endometriosis, polycystic ovary syndrome (PCOS), premature ovarian insufficiency (POI), and ovarian aging (OA). This review systematically summarizes and critically evaluates the current body of research. Furthermore, existing therapeutic strategies targeting mitophagy are classified, the current state of research progress is discussed, and potential future directions are proposed. This comprehensive approach aims to provide novel insights into the treatment of diseases affecting the female reproductive system while improving reproductive health outcomes in women.

## Mitochondrial quality control and mitophagy

Mitochondrial quality control involves maintaining the dynamic balance of mitochondrial fission and fusion, repairing mitochondrial DNA (mtDNA) mutations, and executing its core mechanism, mitophagy, to ensure the functional integrity of the mitochondrial network (14). Mitochondrial dysfunction may occur during cellular differentiation, hypoxic responses, or paternal mtDNA elimination after fertilization. Upon detecting mitochondrial damage, cells regulate mitochondrial distribution and morphology through fusion and fission while activating the mitochondrial unfolded protein response (UPRmt) to address the accumulation of misfolded proteins and restore intracellular homeostasis. However, when these mechanisms fail to adequately restore mitochondrial function, mitophagy selectively targets and degrades damaged mitochondria, thereby preserving mitochondrial quality and maintaining cellular homeostasis (15–18). The process of mitophagy, from its initiation to the clearance of dysfunctional mitochondria, can be divided into four steps (19): 1) A significant loss of mitochondrial membrane potential (MMP/ΔΨm) in the damaged mitochondria. 2) Complete engulfment of mitochondria by autophagosomes, forming mitophagosomes. 3) Fusion of mitophagosomes with lysosomes. 4) Formation of autolysosomes or translocation of lysosomal acid hydrolases into autophagosomes for the degradation of damaged mitochondria. Concurrently, new proteins and lipids are synthesized and integrated into the existing mitochondrial network. With a few exceptions, such as the development of mature lens fiber cells in vertebrates (20), mitophagy and mitochondrial biogenesis are two opposing yet complementary processes that synergistically mediate mitochondrial renewal at multiple levels to restore mitochondrial function.

## The molecular mechanism of mitophagy

The molecular mechanisms of mitophagy are broadly categorized into ubiquitin-dependent and ubiquitin-independent pathways (21). The prototypical ubiquitin-dependent pathway involves the PTEN-induced putative kinase 1 (PINK1)/PARK2 gene-encoded protein (Parkin) signaling cascade, which has emerged as a focal point in studies of mitophagy during female reproduction. Under physiological conditions (22–24), PINK1 is translocated to polarized mitochondria via the outer membrane translocase and inner membrane translocase complex. Upon crossing the inner mitochondrial membrane (IMM), PINK1 undergoes dual cleavage by the phosphoglycerate mutase family member 5-related rhomboid protease within the IMM. This generates an N-terminal fragment containing phenylalanine 104, which is retrotranslocated to the cytosol and subsequently degraded via the N-end rule pathway in a proteasome-dependent manner. This process ensures that PINK1 expression is maintained at low levels in healthy mitochondria. Under pathological conditions (25–28), particularly when cells are exposed to oxidative stress induced by reactive oxygen species (ROS) or other stressors, mitochondrial membrane depolarization prevents PINK1 from being imported into the mitochondria. As a result, PINK1 accumulates on the outer mitochondrial membrane (OMM) and undergoes autophosphorylation at serine 228. Activated PINK1 subsequently phosphorylates ubiquitin at serine 65, generating phosphorylated ubiquitin, which exhibits a high affinity for Parkin and recruits it from the cytoplasm to the mitochondrial surface. Subsequently, PINK1 directly or indirectly phosphorylates Parkin via phospho-Ser65-ubiquitin-mediated recruitment, exposing Parkin’s catalytic domain and triggering its E3 ubiquitin ligase activity. This leads to the formation of extensive ubiquitin chains on proteins on the OMM, serving as substrates for PINK1 and establishing a positive feedback loop that enhances Parkin recruitment and ubiquitination efficiency. Autophagy receptors such as optineurin (OPTN), nuclear dot protein 52 (NDP52), p62, BRCA1-associated protein 1 (NBR1), and TAX1-binding protein 1 (TAX1BP1), which possess ubiquitin-binding domains, recognize and bind to ubiquitinated proteins on damaged mitochondria (29). These receptors interact with Microtubule-Associated Protein 1 Light Chain 3 (LC3) positive autophagosomes, facilitating the selective engulfment and degradation of dysfunctional mitochondria.

In contrast, the ubiquitin-independent pathway primarily relies on receptors that directly interact with LC3 or its homologs. Key mediators of this pathway include the Bcl-2 interacting protein 3-like (BNIP3L/Nix), Bcl-2 interacting protein 3 (BNIP3), and FUN14 domain-containing 1 (FUNDC1) pathways (30, 31). BNIP3L, an OMM protein, mediates mitochondrial fission and mitosis (32, 33) and directly interacts with LC3 on the autophagosomal membrane through its LC3-interacting region (LIR), promoting the engulfment of damaged mitochondria (34, 35). BNIP3 works synergistically with Nix in recruiting Parkin and maintains mitochondrial homeostasis via the PINK1-Parkin pathway (36). FUNDC1, another outer membrane protein harboring an LIR domain, undergoes dephosphorylation at specific residues under hypoxic conditions, enhancing its affinity for LC3 and thereby promoting mitophagy (37). Besides, FUNDC1 synergizes with UNC-51-like kinase 1 (ULK1), leading to Ser17 phosphorylation and enhancing mitophagy activity (38).

## Mitophagy and female reproductive processes

Mitophagy plays a pivotal role in regulating multiple cellular processes during female reproductive physiology, including folliculogenesis, oocyte fertilization, and embryo implantation. The regulatory function of mitophagy can be categorized into four distinct developmental stages: primordial follicles, primary follicles, secondary follicles, and mature follicles. Within primordial follicles, anti-Müllerian hormone (AMH), secreted by granulosa cells, can inhibit interaction with mitophagy. During the transition from primordial to primary follicles, mitophagy may synergize with mitochondrial fusion and fission mechanisms to facilitate oocyte maturation; however, this hypothesis requires further experimental validation. In secondary follicles, mitophagy prevents lipid peroxidation via its substrate Sirtuin 1 (SIRT1), while another sirtuin, SIRT5, suppresses ULK1 phosphorylation, thereby maintaining mitophagy homeostasis and preserving mitochondrial function and oocyte quality. As follicles mature, mitophagy ensures oocyte maturation by suppressing the expression of growth arrest-specific gene 6 (Gas6). However, excessive mitophagy can trigger follicular atresia by exacerbating granulosa cell apoptosis. During fertilization and implantation, mitophagy mediates the elimination of paternal mitochondria, subsequently promoting oocyte protein degradation, modulating trophoblast cell function, and participating in placental angiogenesis and vascular remodeling. The precise role of mitophagy in these later developmental stages remains to be fully elucidated. Finally, we examine the hormonal regulation of mitophagy and its implications for reproductive physiology.

### The genesis and development of follicles

#### Primordial follicle

Primordial follicles constitute the most fundamental follicular structure in the ovary, comprising an oocyte arrested at the diplotene stage of meiosis I and a surrounding monolayer of flattened granulosa cells (GCs) (39). These structures form during embryonic development but predominantly remain in a quiescent or atretic state throughout a person’s life. It is estimated that the number of primordial follicles declines from 2 million at birth to 400,000 by the time of menarche (40). The secretion of AMH by GCs in preantral and small antral follicles (AFs) acts as a critical inhibitory factor of primordial follicle activation. Research by Zhang et al. observed that AMH exerts an inhibitory effect on forkhead box protein O3a, an upstream effector of the PINK1-Parkin pathway (41). This finding indicates a potential link between mitophagy and activation of primordial follicles.

#### Primary follicle

During the growth phase, the oocyte within the primordial follicle exhibits an increase in volume, while the surrounding GCs transition from a flattened to a cuboidal or columnar shape and differentiate into 5–6 layers. At this stage, the primordial follicle develops into a primary follicle (42). To date, there is no direct evidence indicating that the mitophagy pathway regulates the formation of primary follicles. However, Yamada et al. demonstrated that PINK1 and the mitochondrial fusion protein mitofusin 1 work synergistically to maintain the balance between mitochondrial fission and fusion within follicles. This process ensures the maintenance of mitochondrial quality and quantity, thereby facilitating oocyte development and maturation (43, 44). Given that mitochondrial morphology and abundance serve as key biomarkers of cell function, the importance of mitophagy in maintaining mitochondrial quality, as well as its role during primary follicle formation, warrants further investigation.

#### Secondary follicle

The follicle with the lowest hormone threshold develops into a dominant follicle, exhibiting continuous volume expansion. The follicular cells proliferate to form 6–12 layers, and the follicular antrum, along with the cumulus oophorus, begins to emerge. GCs surrounding the follicular antrum form the follicular wall, while the theca differentiates into inner and outer layers, thereby establishing secondary follicles. Sirtuin 1 (SIRT1) is a nicotinamide adenine dinucleotide (NAD+) dependent deacetylase and a substrate of mitophagy (45). SIRT1 agonists can reduce the lipid content in porcine secondary follicles cultivated *in vitro* and prevent lipid peroxidation (46). Furthermore, SIRT1 serves as a crucial regulatory factor of the UPRmt. When cells activate the UPRmt pathway for protein folding and the process fails, this may subsequently trigger protein degradation mechanisms or ultimately induce mitophagy (47). Hence, during the developmental process of secondary follicles, there might exist a correlation between mitophagy and the SIRT-related UPRmt pathway. Sirtuin 5 (SIRT 5), another member of the SIRT family, is localized in mitochondria and plays a critical role in regulating spindle assembly and chromosome alignment during meiosis. This function provides the energy required for biochemical reactions and structural transformations in developing oocytes, thereby promoting oocyte maturation in mice. Inhibition of SIRT5 induces ULK1 phosphorylation and disrupts the balance of the Parkin-dependent mitophagy pathway, resulting in an inability to suppress excessive mitochondrial clearance. Consequently, this leads to mitochondrial dysfunctions, redox impairments, and ultimately compromised oocyte quality. Therefore, Parkin-mediated mitophagy may represent potential therapeutic targets for SIRT5 to enhance oocyte quality and address reproductive disorders associated with mitochondrial dysfunction (48).

#### Mature follicle

Mature follicles, known as AFs, develop from secondary follicles. During this process, the follicular antrum enlarges, the granulosa cell layer thins, and a fluid-filled cystic structure forms. As development progresses, the AF moves closer to the ovarian surface and protrudes outward (49, 50). The maturation of oocytes requires the coordinated development of both cytoplasm and nucleus. The oocyte growth arrest-specific gene 6 (Gas6) is essential for pronucleus formation during oocyte maturation. A deficiency in Gas6 not only impedes oocyte maturation but is also closely linked to the accumulation of dysfunctional mitochondria within the cytoplasm. *In vitro* studies on mice have demonstrated that silencing Gas6 expression suppresses mitophagy, thereby causing impaired cytoplasmic maturation and mitochondrial dysfunction (51). Consequently, Gas6 may play a critical role in promoting oocyte cytoplasmic maturation and maintaining mitochondrial function through the regulation of mitophagy. Furthermore, a significant depletion of RAD51 recombinase 1 in oocytes activates mitophagy, which leads to a decrease in mtDNA copy number and the emergence of mitochondrial dysfunctions. Finally, cytoplasmic maturation of oocytes is inhibited (52). This evidence clearly suggests a close link between the stimulatory role of RAD51 recombinase 1 in the cytoplasmic maturation of oocytes and mitophagy.

#### Atretic follicle

**Figure 1:**
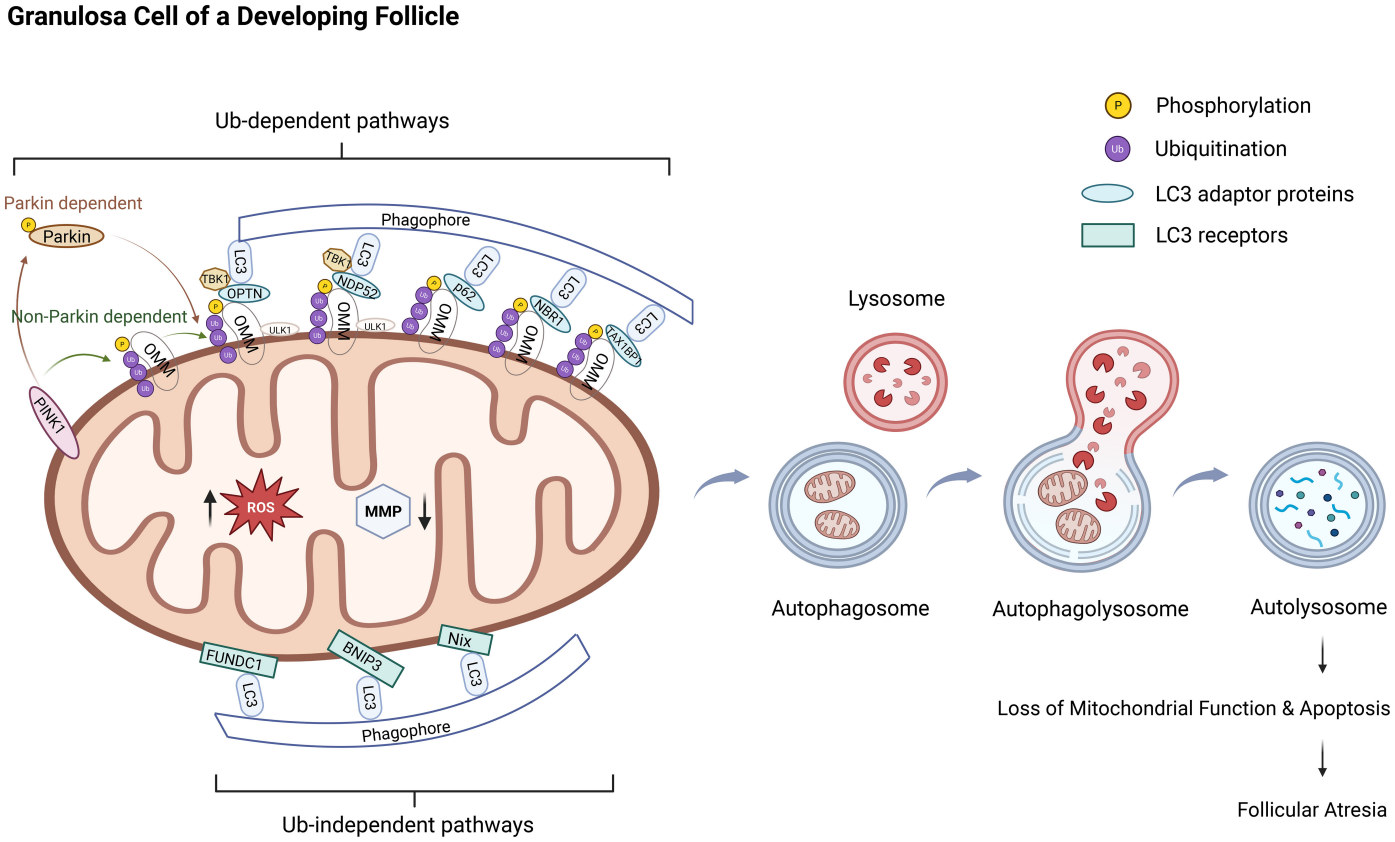
Excessive mitophagy in granulosa cells leads to follicular atresia. The molecular mechanism and progression of mitophagy involve two major pathways: the ubiquitin-dependent pathway and the ubiquitin-independent pathway. The PINK1/Parkin pathway represents a canonical example of the ubiquitin-dependent mitophagy mechanism. Under pathological conditions, such as oxidative stress, mitochondrial membrane depolarization results in the stable accumulation of PINK1 on the outer mitochondrial membrane, where it undergoes autophosphorylation and becomes activated. Activated PINK1 phosphorylates ubiquitin molecules, which facilitates the recruitment of cytosolic Parkin to the mitochondrial surface and activates its E3 ubiquitin ligase activity through phosphorylation. Parkin then catalyzes the assembly of ubiquitin chains on mitochondrial surface proteins, thereby labeling damaged mitochondria for selective degradation and establishing a positive feedback loop that amplifies the mitophagy signal. Ubiquitin-binding autophagy receptors, including OPTN, NDP52, p62, NBR1, and TAX1BP1, recognize these ubiquitinated proteins and bridge the interaction with LC3-positive autophagosomal membranes, thereby mediating the selective engulfment and lysosomal degradation of impaired mitochondria. In contrast, the ubiquitin-independent pathway primarily relies on the direct interaction between mitochondrial outer membrane proteins, such as Nix, BNIP3, and FUNDC1, and LC3 or its homologs to initiate mitophagy. The overall process of mitophagy can be delineated into four sequential stages: initiation, sequestration of dysfunctional mitochondria via autophagosome formation, fusion of autophagosomes with lysosomes, and degradation of mitochondrial components within autolysosomes to ensure the elimination of damaged mitochondria. This schematic illustrates the pathogenic cascade through which excessive mitophagy, driven by oxidative stress, triggers follicular atresia in ovarian granulosa cells. The dysregulation of this quality-control mechanism leads to uncontrolled mitochondrial clearance, culminating in bioenergetic failure and the initiation of apoptosis. The subsequent extensive loss of granulosa cells directly drives follicular degeneration.Ub: ubiquitin, Parkin: PARK2 gene-encoded protein, PINK1:PTEN-induced putative kinase 1, ROS: reactive oxygen species, MMP/ΔΨm: mitochondrial membrane potential, LC3: Microtubule-Associated Protein 1 Light Chain 3, OMM: outer mitochondrial membrane, OPTN: optineurin, NDP52: nuclear dot protein 52, SQSTM1/P62: sequestosome-1, NBR1: BRCA1-associated protein 1, TAX1BP1: TAX1-binding protein 1, TBK1:TANK-Binding Kinase 1, BNIP3L/Nix: Bcl-2 interacting protein 3-like, BNIP3: Bcl-2 interacting protein 3, FUNDC1: FUN14 domain-containing 1.

**Figure 2:**
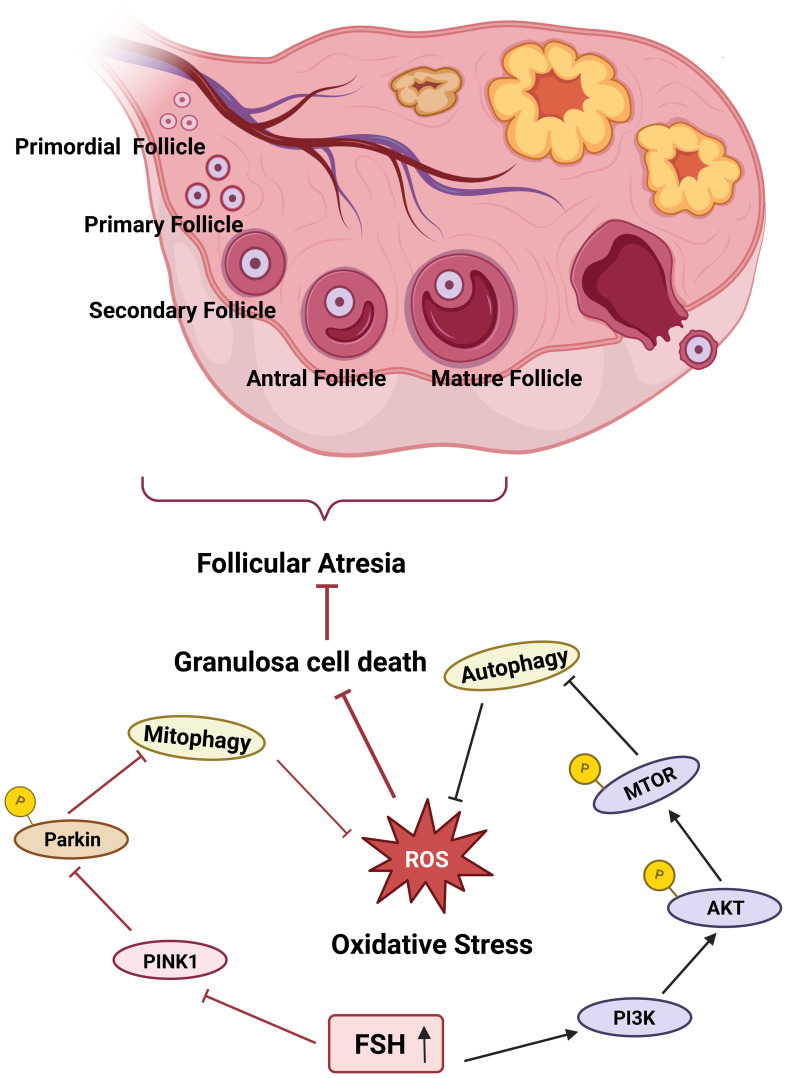
FSH suppresses mitophagy to prevent follicular atresia. FSH prevents follicular atresia by deploying a dual strategy in granulosa cells. Activation of the PI3K-AKT-mTOR pathway suppresses autophagic activity, while inhibition of the PINK1-Parkin pathway dampens mitophagy, thereby alleviating oxidative damage and preventing excessive mitochondrial loss. This coordinated regulation thus enhances granulosa cell vitality and forestalls the onset of atresia.

At the end of the ovarian cycle, most follicles that do not undergo ovulation eventually undergo atresia and degenerate, with 99% of all follicles being subject to this process (53). In mammalian embryos, atretic follicles begin to develop as early as six weeks of gestation *in utero*. Atresia can occur at any stage of follicular development; however, it is most prevalent during the AF stage. The apoptosis of GCs represents the primary cause of follicular atresia. Oxidative stress plays a critical role in GC-induced follicular atresia. Follicle-stimulating hormone (FSH) protects GCs, which are highly sensitive to ROS, from undergoing apoptosis and reduces GC mortality. Specifically, FSH inhibits PINK1 expression, prevents Parkin translocation to mitochondria, suppresses excessive mitophagy activation, and thereby maintains GC viability (54) (**Figure 1**). Furthermore, FSH can activate the phosphoinositide 3-kinase (PI3K)-AKT-mechanistic target of rapamycin (MTOR) pathway. The PI3K-AKT-MTOR pathway is a crucial pathway for the downregulation of autophagy. By suppressing excessive autophagy, it protects GCs in an oxidative damage milieu and facilitates ovulation (**Figure 2**). Mitophagy exhibits bidirectional regulatory properties. In this respect, Li et al. demonstrated that under hypoxic conditions, FSH activates the mitophagy pathway, leading to upregulated expression of Parkin, PINK1, and PTEN, which in turn inhibits germ cell apoptosis. This indicates that mitophagy is not merely a simple on/off mechanism. In the context of female reproduction, the tissue-specific characteristics and molecular signaling intensity thresholds associated with the bidirectional regulation of mitophagy warrant further investigation, as they may be closely linked to the clinical therapeutic window for reproductive disorders (55).

### Fertilization and implantation

The process of sperm-egg fertilization is associated with the elimination of specific reproductive organelles. In many species, selective autophagy mediates the degradation of paternal mitochondria following fertilization while preserving maternal mitochondria. In *C. elegans*, the elimination of paternal mitochondria is mediated through ubiquitination and the mitophagy pathway, with the mitophagy process being directly regulated by FUNDC1 (56, 57). The phenomenon of paternal mitochondrial elimination has also been observed in mice. Upon the sperm reaches the oviduct, most of its mitochondria have already undergone phagocytosis and degradation (58). The strict maternal inheritance of mitochondria in mice is dependent on the interplay between mitochondrial E3 ubiquitin protein ligase 1 and the Parkin-mediated mitophagy pathway (59). In early embryos, the autophagy mechanism mediates the degradation of oocyte proteins, thereby facilitating embryonic implantation (60). At the blastocyst stage, trophoblast cells are regulated by autophagy, which promotes normal placental development (61). Autophagy collaborates with C-X-C chemokine ligand 12 and its receptors to participate in placental angiogenesis and vascularization, maintaining placental homeostasis (62). The key autophagy factors ATG5 and BECN1 play essential roles in embryonic organogenesis and development (63, 64). Collectively, autophagy is involved in a series of developmental processes, including pre-implantation, implantation, and post-implantation stages of embryogenesis. However, whether mitophagy directly modulates fertilization and implantation during female reproductive processes remains to be elucidated. According to current studies (65, 66), mitophagy may sustain cellular energy metabolism and oxidative stress balance, thereby providing adequate energy support for blastocyst cell migration, embryo adhesion, and embryogenesis. The precise mechanisms underlying this biological process warrant further investigation.

### Sex hormones

As previously discussed, follicular atresia is regulated by both apoptosis and mitophagy, with FSH serving as a key regulatory factor linking these two mechanisms. FSH modulates granulosa cell activity by either activating or suppressing excessive mitophagy under varying redox conditions. Furthermore, high-dose FSH has been shown to induce autophagy in bovine granulosa cells via the AKT-MTOR signaling pathway, thereby enhancing estradiol (E_2_) production (67). In a study involving porcine oocytes, E_2_ was found to alleviate oxidative stress, inhibit apoptosis, and promote *in vitro* maturation and developmental competence through autophagy-related mechanisms (68). These findings collectively indicate that FSH plays a pivotal role in mitophagy in the context of improving female reproductive function. During follicular development, AMH, secreted by granulosa cells of preantral and small antral follicles in the ovary, has been shown to inhibit forkhead box O3a (FOXO3a), an upstream effector of the PINK1-Parkin-mediated mitophagy pathway. This suggests that mitophagy may be involved in follicular activation; however, direct experimental evidence is required to confirm this hypothesis. Mitochondrial uncoupling protein 2 (UCP2), a mitochondrial membrane protein, contributes to mitochondrial homeostasis by reducing ROS, regulating apoptosis, and maintaining calcium homeostasis. In human cumulus cells, UCP2 has been implicated in the regulation of ROS production, apoptosis, and progesterone synthesis via autophagy, thereby participating in follicular development and early embryo implantation (69). Given the close interplay between mitophagy, oxidative stress, and apoptosis, it is of scientific interest to investigate whether UCP2 interacts with mitophagy and whether such interaction contributes to reproductive function. This warrants further experimental exploration.

## Mitophagy and female reproductive disorders

Mitophagy is closely associated with the onset and progression of diseases affecting the female reproductive system, including endometriosis, PCOS, POI, and OA (**Table 1**). In the context of endometriosis, mitophagy plays a regulatory role in the apoptosis and migration of endometrial stromal cells, thereby suppressing the formation of ectopic implantation lesions. Regarding PCOS, mitophagy primarily affects ovarian granulosa cells. Excessive activation of mitophagy may impair granulosa cell function, as evidenced by reduced expression of MMPs and mtDNA. The administration of Reverse Erythroblastosis Virus Oncogene Homolog (REV-ERB) has been shown to ameliorate such cellular dysfunction. Furthermore, iron-dependent mitophagy operates under varying oxidative conditions within granulosa cells, contributing to improved follicular development. The role of mitophagy in POI is complex. Notably, increased levels of autophagosomes and autolysosomes have been observed in granulosa cells of POI mouse models, along with elevated expression of mitophagy-related proteins in ovarian tissues compared to normal controls. Conversely, some studies have suggested that enhanced mitophagy activity may promote follicular development and regulate sex hormone levels, thereby improving ovarian function; however, the underlying mechanisms require further investigation. Finally, in OA, mitophagy primarily influences the meiotic progression of germinal vesicle-stage oocytes, helping to correct maturation defects. Suppression of hyperactivated mitophagy has been shown to enhance oocyte quality in OA.

**Table 1:** Mitophagy and reproductive disorders.

Reproductive disorder	Mitophagy contribution	Reference
Endometriosis	PINK1-Parkin-mediated mitophagy inhibits cell proliferation, migration and invasion, and enhances apoptosis. REV-ERB directly acts on the mitochondrial quality control system.	(70) (71)
PCOS	REV-ERB agonists attenuate the excessive activation of mitophagy in mice with PCOS, promote the normal development of follicles.	(72, 73)
	An elevation in iron levels triggers the activation of the TFRC/NOX1/PINK1/ACSL4 pathway, consequently impairing the normal development of follicles in PCOS mice and KNG cells.	(74)
	Inhibition of CISD2 expression in PCOS patients and testosterone-treated mice activates the PINK1-Parkin pathway, preserves the stability of the follicular microenvironment.	(75)
	Melatonin inhibits the over-activated PINK1/Parkin mitophagy pathway in DHT-treated KGN cells and mice, as well as in PCOS patients, thereby alleviating granulosa cell damage in PCOS.	(76, 77, 78)
POI	Overexpression of Nur77 in the ovaries of mice with POI induces activation of the PINK1-Parkin pathway, thereby enhancing follicular development and restoring sex hormone levels.	(79)
OA	RAB7 suppresses excessive PINK1-Parkin-mediated mitophagy, thereby enhancing the quality of oocytes in mice with OA.	(80)
	CNP suppressed excessive mitophagy in the oocytes of OA mice, mitigated DNA damage and apoptosis, and provided adequate time for cytoplasmic maturation.	(81, 82)
	Spermidine enhances oocyte quality through the activation of mitophagy.	(83)
	Rg1 induces the activation of t-BHP-mediated mitophagy in Drosophila and mitigates reproductive damage associated with oxidative stress.	(84)
	Salidroside activates mitophagy, thereby enhancing mitochondrial function and alleviating oxidative damage in oocytes of OA mice.	(85)

### Endometriosis

Endometriosis is a chronic, hormone-dependent inflammatory disorder characterized by the ectopic implantation of endometrial glands and stroma outside the uterine cavity. It has been established as one of the leading causes of pelvic pain, dysmenorrhea, and infertility, affecting approximately 5% to 10% of women of reproductive age (86, 87). Accumulating evidence indicates that the interplay among apoptosis, angiogenesis, autophagy, and mitophagy plays a complex role in the pathogenesis of endometriosis in rodent models (88). PINK1 serves as a key initiator of mitophagy. In rat models of endometriosis (70), the PINK1-Parkin-mediated mitophagy pathway suppresses cell proliferation, migration, and invasion through upregulation of prohibitin 2. Furthermore, the PINK1-Parkin mitophagy pathway represents a critical mechanism by which macrophage stimulator 1 (Mst1), a negative regulator of endometriosis, modulates apoptosis and migration in human endometrial stromal cells (ESCs) (89). Mst1 inhibits Parkin transcription and expression, thereby suppressing mitophagy and promoting ESC apoptosis while restricting cell migration. Specifically, Mst1 overexpression leads to reduced Parkin expression, mitochondrial fragmentation, impaired lysosomal co-localization, cytoplasmic calcium overload, and decreased F-actin expression. Besides, the coordinated regulation of mitophagy and apoptosis via the PINK1-Parkin pathway has been associated with the mTOR signaling cascade (90, 91). Both autophagy and PINK1-Parkin-mediated mitophagy activate the mTOR pathway, which in turn stimulates pro-apoptotic Bcl-2 family proteins on the mitochondrial membrane, ultimately inducing apoptosis through calcium channel blockers. Endometrial cell apoptosis can counteract angiogenesis to some extent, thereby reducing the volume, area, and diameter of endometriotic lesions and impeding disease progression.

The mitochondrial quality control system in endometriosis has been closely linked to REV-ERB. Brain and muscle aryl hydrocarbon receptor nuclear translocator-like 1 (BMAL1) and Circadian Locomotor Output Cycles Kaput (CLOCK) are core components of the circadian clock machinery. REV-ERB forms a feedback regulatory loop with downstream target genes, directly influencing mitochondrial quality control and sleep-wake patterns (71). The chronic estrogen dependence of endometriosis may further contribute to sleep disturbances. Modulating circadian rhythms and restoring mitochondrial function may offer therapeutic potential for endometriosis. Although direct evidence linking mitophagy to circadian regulation remains limited, mitochondrial fission and fusion, processes closely associated with mitophagy, exhibit circadian oscillations synchronized with the light/dark cycle through the phosphorylation-dependent activation and inactivation of DRP1 (92). These findings suggest that mitophagy may play a role in the regulation of circadian rhythms and the progression of endometriosis.

### PCOS

PCOS is the most prevalent endocrine disorder in women of reproductive age, with major characteristics encompassing ovulation dysfunction, hyperandrogenism and polycystic ovarian morphology. One in six women of reproductive age is afflicted by PCOS, which constitutes the primary cause of subfertility (93, 94). The pathogenesis of PCOS is closely linked to mitochondrial energy metabolism (95). Studies have shown that in human ovarian granulosa cells (KNG) treated with dihydrotestosterone, the MMP and mtDNA content were reduced, whereas the abundance of autophagosomes and the levels of key mitophagy proteins PINK1 and Parkin were elevated. This suggests that excessive activation of mitophagy contributes to GC damage. Comparable alterations were detected in the GCs of individuals diagnosed with PCOS. In addition, the study by Zhao et al. revealed that in the GCs of patients with PCOS, in addition to the previously mentioned alterations, the mitophagy receptors Nix and RHEB were also highly expressed. This finding provides further evidence of the involvement of mitophagy in granulosa cell dysfunction in PCOS. Dysfunctional GCs can induce oxidative stress and chronic inflammation, consistent with the pathophysiological mechanisms underlying PCOS (96, 97). Future studies should aim to directly validate this targeted relationship, thereby facilitating potential clinical translation.

REV-ERB serves as a central regulator of the circadian clock and is intricately linked to mitochondrial biosynthetic functions (98). REV-ERB inhibits the translocation of Park2, a key factor in mitophagy, to mitochondria or modulates the activity of the mitophagy activator ULK1, thereby maintaining mitochondrial structure and function (72). In PCOS patients, REV-ERB expression is significantly reduced in GCs (73). A study by Amador et al. found that treating PCOS mice with SR9009, a REV-ERB agonist, suppressed over-activated mitophagy. This upregulated the expression of peroxisome proliferator-activated receptor γ coactivator 1α, nuclear respiratory factor 1, and mitochondrial transcription factor A, genes associated with mitochondrial biogenesis. The enhanced expression of these genes promoted mitochondrial biogenesis, corrected quality defects in GCs caused by PCOS, and facilitated follicular development and maturation, thereby regulating female reproductive capacity.

Iron-mediated mitophagy plays a critical role in the pathogenesis of PCOS. Ammonium ferric citrate activates transferrin receptor 1 (TFRC), thereby increasing intracellular iron levels, which leads to the accumulation and release of ROS, overactivation of the PINK1-dependent mitophagy pathway, induction of ferroptosis, and inhibition of follicular development via the TFRC/NOX1/PINK1/ACSL4 signaling axis. Therefore, reducing iron uptake may facilitate normal follicular development in PCOS. Zhang et al. (74) consistently reported that activation of ACSL4 in KNG cells suppressed normal follicular development, with abnormal follicle formation being closely associated with the initiation and progression of PCOS (99). The regulatory mechanism of mitophagy is influenced by the cellular redox status. CDGSH iron-sulfur domain 2 (CISD2), a protein found on the outer mitochondrial membrane, endoplasmic reticulum, and mitochondria-associated membranes, functions as a [2Fe-2S] cluster-containing protein with oxygen-reducing activity, and participates in the regulation of cellular iron metabolism, ROS homeostasis, and mitophagy (100–103). The involvement of CISD2 in electron and iron-sulfur cluster transfer defines its functional relationship with mitophagy. Under reducing conditions, CISD2 is unable to transfer [2Fe-2S] clusters; however, under oxidative stress, CISD2 facilitates iron transport into the mitochondrial matrix and transfers electrons to oxygen through oxidized NAD+ (an electron donor), thereby promoting oxidative stress and generating superoxide radicals (O_2_^−^) (104–106). Wu et al. (75) established a PCOS model in KNG cells using testosterone and observed elevated CISD2 expression under oxidative conditions. Silencing CISD2 expression via shRNA significantly enhanced PINK1-Parkin-mediated mitophagy and upregulated SOD2 expression, thereby attenuating oxidative stress and stabilizing the follicular microenvironment.

Besides, studies have explored PCOS-related clinical features such as insulin resistance and obesity. In obese humans and rats, the expression levels of mitophagy-related molecules, including Parkin, FUNDC1, and BNIP3, are markedly decreased. These mitophagy defects impair the metabolic differentiation of adipose tissue, contributing to insulin resistance (76–78). Therefore, mitophagy may alleviate PCOS symptoms by modulating metabolic pathways, offering valuable insights for developing clinical strategies.

### POI

POI, characterized by the premature depletion of ovarian follicles before the age of 40, is a major contributor to female infertility (107). Mitophagy plays a critical role in maintaining ovarian function by mitigating excessive ROS accumulation and preventing mtDNA damage. However, excessive activation of mitophagy may contribute to the development of POI. Miao et al. demonstrated that in POI mice, the expression levels of PINK1 and Parkin were elevated in the ovaries, accompanied by an increased number of autophagosomes and autolysosomes in GCs, suggesting that excessive mitophagy is involved in the pathogenesis of POI (108). A cohort study involving 375 patients identified mitophagy as a potential therapeutic target for POI (109). Sequencing analysis revealed a homozygous single-nucleotide insertion in exon 1 of the SPATA33 gene (NM_153025.2: c.34dup; p.Cys12LeufsTer2). SPATA33, a protein exclusively expressed in mitochondrial germ cells, has been recognized as a novel mediator of mitophagy (110), providing genetic evidence linking POI with mitophagy. There are varying perspectives on the precise relationship between mitophagy and the pathogenesis of POI. Some studies have indicated that the pathological process of POI is closely associated with the suppression of the PINK1-Parkin pathway (111, 112). Yao et al. overexpressed neurotrophin-induced gene B (Nur77) in the ovaries of POI mice. Nur77, a member of the nuclear hormone receptor NR4A family, regulates pathological processes such as metabolic abnormalities, hypoxia stress, and inflammation. Activation of Nur77 can induce PINK1-Parkin pathway-mediated mitophagy, thereby improving follicular development and sex hormone levels in POI mice and enhancing ovarian function. However, this study lacked a control group with inhibited mitophagy and did not include recovery experiments following Nur77 overexpression (79). Compared to other reproductive disorders, research on POI is extensive and diverse, offering multiple perspectives for future investigations and aiding in the elucidation of the precise mechanisms of mitophagy’s involvement.

### OA

OA is defined as the progressive decline and ultimate exhaustion of ovarian function, marked by a reduction in follicle abundance and deterioration in oocyte quality (13). Furthermore, compromised oocyte quality is strongly associated with adverse reproductive outcomes, such as fertilization failure, impaired embryo development, and miscarriage. Wang et al. demonstrated that excessive activation of mitophagy mediated by the PINK1-Parkin pathway plays a critical role in the physiological mechanisms underlying OA, particularly in germinal vesicle-stage oocytes (113). Pan et al. proposed that both mitophagy and mitochondrial trafficking contribute to OA. Specifically, Parkin, lysosome-associated membrane protein 2, and mitochondrial dynamics-related protein 1 worked synergistically with mitochondrial Rho-GTPase, an OMM protein, to regulate mitochondrial transport and mitophagy during oocyte meiosis, thereby alleviating maturation defects in OA oocytes (114). Jin et al. further revealed that mitophagy exerts regulatory effects during this process. Notably, RAB7, a key regulator of the late endosome/lysosome network, remains active during meiosis to suppress excessive PINK1-Parkin-mediated mitophagy, thus enhancing oocyte quality in the context of OA (80). Besides, endogenous C-type natriuretic peptide (CNP), secreted by GCs in the follicular wall, mitigates DNA damage and apoptosis in OA oocytes, ensuring adequate time for cytoplasmic maturation (81, 82). This effect is primarily achieved through CNP destabilizing PINK1 and inhibiting Parkin recruitment, thereby restoring mitochondrial oxidative phosphorylation. The therapeutic potential of compounds like spermidine and traditional Chinese medicine for OA will be discussed in more detail later.

## Targeted mitophagy-based therapeutic strategies

In addition to the aforementioned strategies, both molecular compounds and TCM have shown considerable promise for enhancing female reproductive function. Key molecular compounds include melatonin, zinc, spermine, and prostaglandin F2α (PGF2α). Current evidence suggests that melatonin modulates granulosa cell function through activation of the mitophagy pathway, specifically by mitigating oxidative stress-induced damage and apoptosis. Zinc and spermine exert beneficial effects on oocyte mitochondrial function through distinct regulatory mechanisms: zinc suppresses excessive mitophagy activation, whereas spermine promotes mitophagy activity, ultimately contributing to improved oocyte quality. PGF2α plays a crucial role in initiating luteolysis by activating mitophagy during the early phase of corpus luteum regression, which may help ameliorate luteal insufficiency in women. In the context of TCM, ginsenoside Rg1 and salidroside have been demonstrated to enhance mitochondrial function in oocytes and improve overall reproductive capacity.

### Molecular compound

Melatonin (N-acetyl-5-methoxytryptamine) is a compound secreted in the ovary, composed of indoleamine and acetyl groups. Its receptors, MT1 and MT2, are highly expressed in GCs, thereby enhancing cellular communication between GCs and melatonin. This not only supports the physiological functions of GCs but also amplifies melatonin’s regulatory effects on GCs (115–117) Several studies (118, 119) have confirmed that during female reproduction, melatonin mitigates oxidative damage in GCs, reduces cell apoptosis, and promotes oocyte maturation. Xu et al. (120) proposed that melatonin regulates GCs via mitophagy. Specifically, melatonin upregulates the expression of PINK1, Parkin, BECLIN1, and LC3II/LC3I in bovine GCs, activating PINK1-Parkin-mediated mitophagy and enhancing reproductive capacity. The activation of mitophagy by melatonin relies on the SIRT1-FoxO1 signaling pathway. More precisely, melatonin interacts with NAD+-dependent histone deacetylase SIRT1, which deacetylates FoxO1 and inhibits its activity (121, 122), thereby reducing the transcriptional activity of pro-apoptotic factors mediated by FoxO1, decreasing GC apoptosis, and improving follicular development (123). In the context of PCOS, mitophagy exhibits protective effects on GCs through diverse regulatory mechanisms (124). In KNG cells, mice, and PCOS patients, melatonin significantly increases SIRT1 expression, suppresses excessive activation of the PINK1-Parkin pathway, restores mitochondrial function, and alleviates GC damage caused by PCOS, thereby improving both *in vivo* and *in vitro* phenotypes of PCOS. Therefore, further investigation into the mechanism of melatonin’s effects on GCs through mitophagy in various cellular environments is warranted.

Research (125, 126) on porcine oocytes has demonstrated that excessive activation of PINK1-Parkin-mediated mitophagy can lead to zinc deficiency. Zinc is an essential trace element that plays a critical role in numerous cellular physiological processes, including transcription, protein synthesis, enzyme activity, cell division, growth, and transport. In the female reproductive system, zinc deficiency inhibits the synthesis and activity of copper-zinc SOD2, increases the acetylation level of SOD2, and enhances cellular sensitivity to ROS, thereby triggering oxidative stress and early apoptosis in oocytes. These cellular events impair meiotic progression, disrupt cytoskeletal integrity, and cause mitochondrial dysfunction, ultimately reducing oocyte quality. Therefore, zinc supplementation may serve to inhibit hyperactivated mitophagy, alleviate oxidative stress, restore mitochondrial function in oocytes, and thereby maintain intracellular homeostasis within oocytes. Selenium is an essential trace element for female reproductive health. It is predominantly localized in the granulosa cell layer and highly expressed in large, healthy follicles, where it may serve as an antioxidant during the later stages of follicular development (127). Given the bidirectional regulatory relationship between mitophagy and oxidative stress, it is essential to investigate the involvement of mitophagy in selenium-mediated regulation of the female reproductive system. Furthermore, Zhang et al. (83) confirmed through non-targeted metabolomics technology that spermidine, a polyamine metabolite, is a key metabolite in the ovary. Increasing the level of spermidine in the ovaries of aged mice promoted follicular development, oocyte maturation, and early embryo development. Microtranscriptomic studies further revealed that this improvement in oocyte quality was achieved through activation of mitophagy and mitochondrial function mediation, and this mechanism remains active under oxidative stress conditions in porcine oocytes. In summary, regulating the mitophagy pathway effectively enhances oocyte quality.

Luteolysis is a pivotal regulatory mechanism in the female reproductive cycle. Bennegard et al. (128) suggested that elucidating the cellular events occurring during early luteolysis could be an important strategy for improving female fertility. Auletta et al. demonstrated that increasing PGF2α levels in the rhesus monkey luteum could induce luteolysis (129). Similarly, Plewes et al. observed this phenomenon in bovine luteum (130). During the early stages of luteolysis, PGF2α activates PINK1 and stimulates Parkin phosphorylation. This finding suggests that mitophagy and mitochondrial fission are involved in the early cellular activities of luteolysis. Although the PGF2α analogues used in these studies do not fully replicate the PGF2α secreted by the uterus, and physiological luteolysis involves more complex mechanisms than PGF2α signaling alone, this research highlights the potential of mitophagy as a therapeutic target for luteal insufficiency. The aforementioned therapeutic approaches targeting mitophagy have shown promising effects on granulosa cell damage, oocyte quality defects, and luteal insufficiency. Thus, mitophagy holds significant potential as a therapeutic target for enhancing female reproductive capacity.

### TCM

Ginseng, a perennial herb belonging to the genus *Panax* in the family Araliaceae, contains ginsenoside Rg1 as its primary bioactive constituent. Ginsenoside Rg1 is a tetracyclic triterpene saponin that has demonstrated protective effects against oxidative stress-induced damage in various pathological conditions, including diabetes, ischemic stroke, and depression (131–133). In diabetic rat models, ginsenoside Rg1 significantly elevated superoxide dismutase (SOD) levels. In both *in vivo* and *in vitro* models of ischemic stroke, ginsenoside Rg1 activated the Nrf2/ARE signaling pathway, thereby enhancing the cellular antioxidant defense system. Furthermore, ginsenoside Rg1 was shown to downregulate the expression of NADPH oxidase isoforms NOX1 and NOX4 in the hippocampus of depression-induced rats, thereby alleviating oxidative stress. As previously discussed, a bidirectional regulatory relationship exists between oxidative stress and mitophagy. Ginsenoside Rg1 has also been reported to improve fertility in ovarian aging mouse models by increasing antioxidant enzyme levels, suggesting its potential regulatory role in the female reproductive system via mitophagy modulation (134). A study by Yang et al. (84) established an oxidative stress-induced OA model in *Drosophila* using tert-butyl hydroperoxide and demonstrated that ginsenoside Rg1 treatment induced PINK1-mediated mitophagy, thereby reducing oxidative damage and improving reproductive capacity. Molecular docking analysis further revealed that Rg1 exhibited strong binding affinity with the active domain of PINK1 and formed hydrogen bonds. These findings suggest that ginsenoside Rg1 may exert its therapeutic effects in OA by activating the PINK1-mediated mitophagy pathway in ovarian cells, promoting mitochondrial degradation, reducing excessive ROS accumulation, and alleviating redox imbalance caused by decreased SOD2 and catalase activity, ultimately reversing oxidative stress-induced reproductive damage.

Recent studies have indicated that salidroside (2-(4-hydroxyphenyl)ethyl-β-D-glucopyranoside), the primary bioactive compound extracted from the roots and rhizomes of *Rhodiola rosea*, exhibits therapeutic potential in the treatment of premature ovarian aging. In an experimental study on porcine oocytes (135), salidroside significantly reduced ROS levels, enhanced MMP and ATP production, increased mitochondrial DNA copy number, and promoted both cytoplasmic and nuclear maturation of oocytes. In subsequent embryo development, salidroside-treated embryos exhibited increased blastomere counts, improved blastocyst proliferation, and upregulated expression of pluripotency genes. Moreover, mitochondrial-targeted molecules have been shown to ameliorate spindle and chromosome abnormalities in aged mouse and human oocytes, suggesting that mitochondrial dysfunction plays a central role in the pathogenesis of ovarian aging (136). Therefore, the therapeutic effects of salidroside on OA are closely associated with mitophagy regulation. A recent study (85) confirmed through transcriptomic and microproteomic analyses that salidroside could maintain normal spindle and chromosome alignment and preserve mitochondrial membrane potential via mitophagy activation, thereby enhancing oocyte maturation, fertilization capacity, and embryonic developmental potential in OA mouse models.

Both ginsenoside Rg1 and salidroside are bioactive constituents of TCM, which are characterized by their multi-target and multi-pathway regulatory properties. Salidroside, for instance, interacts with key molecular targets such as Tumor Necrosis Factor-alpha, Interleukin-2, Bcl-2, Cyclooxygenase-2, Vascular Endothelial Growth Factor, cysteine-aspartic acid protease 3, and Hypoxia-Inducible Factor-1alpha, and modulates multiple signaling pathways including PI3K/Akt/mTOR, Mitogen-Activated Protein Kinases, Extracellular Signal-Regulated Kinase 1 and 2, Glycogen Synthase Kinase-3 Beta, and Nuclear Factor Erythroid 2-Related Factor 2. These molecular targets and pathways are closely associated with the pathophysiological mechanisms of female reproductive disorders, underscoring the therapeutic potential of salidroside in reproductive medicine. Despite its promising effects, there remains a lack of comprehensive long-term toxicological data to support its clinical application. However, existing toxicity studies have not identified significant adverse effects. Besides, the bioavailability of salidroside is closely related to its synthetic methodology. Therefore, optimizing the synthesis and derivatization of salidroside represents a promising avenue for advancing its clinical application in TCM (137).

## Conclusions

Mitophagy represents a critical mechanism for mitochondrial quality control, with most current research centered on the PINK1/Parkin signaling pathway. Within the context of female reproductive physiology, mitophagy exerts essential regulatory functions in follicular development and fertilization. Notably, it demonstrates bidirectional regulatory properties during follicular atresia, a phenomenon that is also evident in the pathogenesis and therapeutic strategies of reproductive disorders. The bidirectional regulation is primarily governed by the intracellular redox status. Future investigations should aim to elucidate the biological thresholds that determine mitophagy activation and suppression, as well as the tissue- and cell-specific variations, which may facilitate the development of precise regulatory interventions for female reproductive diseases. Research on mitophagy’s role during embryo implantation and post-implantation development remains limited and primarily indirect. Nevertheless, this area holds significant potential for improving female pregnancy outcomes and advancing assisted reproductive technologies, warranting further in-depth exploration.

In the pathological context of the female reproductive system, mitophagy has been implicated in the progression of endometriosis, PCOS, premature ovarian insufficiency, and ovarian aging. Studies on the regulatory mechanisms of mitophagy have revealed that it not only collaborates with key mitochondrial quality control pathways, including mitochondrial biogenesis, fission/fusion dynamics, and transport, but also interacts with the mitochondrial unfolded protein response (UPRmt), ferroptosis signaling, and apoptotic cascades. The complex interplay between mitophagy and apoptosis is particularly notable across multiple biological levels and processes. During follicular atresia, mitophagy suppresses granulosa cell apoptosis and sustains cellular viability. In endometriosis, it modulates the apoptotic and migratory behaviors of endometrial stromal cells, thereby mitigating lesion progression. In PCOS, inhibition of the PINK1/Parkin pathway reduces oocyte apoptosis and enhances oocyte quality. Besides, melatonin and zinc have been shown to enhance reproductive function by mitigating granulosa and oocyte apoptosis via mitophagy induction. Emerging evidence further suggests that epigenetic mechanisms may directly regulate the mitochondrial quality control network, implying a potential targeted interaction between epigenetic modifications and mitophagy (138). However, the underlying mechanisms linking these processes in the context of reproductive biology remain to be fully elucidated. Collectively, these findings expand our understanding of the molecular regulatory networks involving mitophagy and underscore its therapeutic potential in the prevention and treatment of female reproductive disorders.

Beyond well-characterized compounds such as melatonin, zinc, spermidine, and prostaglandin F2α, bioactive constituents of traditional Chinese medicine, such as ginsenoside Rg1 and salidroside, have demonstrated the capacity to enhance female reproductive function through the modulation of mitophagy. The principal mechanism involves the mitigation of ROS-induced cellular damage. However, current studies on mitophagy in relation to traditional Chinese medicine remain limited in both scope and methodological rigor. Future research should focus on delineating the interplay between mitophagy and multiple molecular signaling pathways, while refining experimental designs to identify and validate specific therapeutic targets. To date, most investigations into mitophagy have been conducted using *in vitro* cell models or *in vivo* animal systems. Advances in high-throughput sequencing technologies and machine learning methodologies offer novel opportunities to integrate multi-omics approaches, identify key regulatory mitophagy factors, and validate their functional roles across experimental platforms, including *in vivo*, *in vitro*, and clinical settings. These developments are critical for establishing the clinical relevance of mitophagy in the diagnosis and therapeutic management of female reproductive disorders.
